# Arterial Stiffening Provides Sufficient Explanation for Primary Hypertension

**DOI:** 10.1371/journal.pcbi.1003634

**Published:** 2014-05-22

**Authors:** Klas H. Pettersen, Scott M. Bugenhagen, Javaid Nauman, Daniel A. Beard, Stig W. Omholt

**Affiliations:** 1 Department of Mathematical and Technological Sciences, Norwegian University of Life Sciences, Ås, Norway; 2 Department of Physiology, Medical College of Wisconsin, Milwaukee, Wisconsin, United States of America; 3 Department of Circulation and Medical Imaging, Cardiac Exercise Research Group, NTNU Norwegian University of Science and Technology, Trondheim, Norway; Johns Hopkins University, United States of America

## Abstract

Hypertension is one of the most common age-related chronic disorders, and by predisposing individuals for heart failure, stroke, and kidney disease, it is a major source of morbidity and mortality. Its etiology remains enigmatic despite intense research efforts over many decades. By use of empirically well-constrained computer models describing the coupled function of the baroreceptor reflex and mechanics of the circulatory system, we demonstrate quantitatively that arterial stiffening seems sufficient to explain age-related emergence of hypertension. Specifically, the empirically observed chronic changes in pulse pressure with age and the impaired capacity of hypertensive individuals to regulate short-term changes in blood pressure arise as emergent properties of the integrated system. The results are consistent with available experimental data from chemical and surgical manipulation of the cardio-vascular system. In contrast to widely held opinions, the results suggest that primary hypertension can be attributed to a mechanogenic etiology without challenging current conceptions of renal and sympathetic nervous system function.

## Author Summary

Hypertension is highly age-related and affects more than 1 billion people worldwide. It is a major source of morbidity and mortality as it makes us more prone to experience heart failure, stroke, and kidney disease. Despite intense research efforts over several decades, there is still no consensus on what are the primary causes of this disorder. Here we present a computational physiology model which shows that the increase in arterial stiffness that follows with age is sufficient to account for an overwhelming amount of experimental and clinical data on hypertension. We demonstrate quantitatively that the stiffening causes the baroreceptors, the blood-pressure sensors located in the arterial wall, to misinform the highly complex machinery responsible for blood pressure regulation. This misinformation occurs because the baroreceptors are strain sensitive, not pressure sensitive, and with stiffening the aortic wall strain ceases to be a good proxy for aortic blood pressure. Contrary to wide-held conceptions, the blood pressure regulation may thus become compromised without any other detrimental physiological change of the regulatory machinery. Our results therefore suggest that arterial stiffness represents a major therapeutic target by which an otherwise intact physiological machinery may be exploited for blood pressure regulation.

## Introduction

The progressive increase in blood pressure with age is characterized by a greater increase in systolic blood pressure than diastolic blood pressure from the middle adult years [1]. While systolic blood pressure continues to rise until the eighth or ninth decade, diastolic blood pressure tends to remain constant or decline after the fifth or sixth decade, leading to an accelerated rise in pulse pressure after age 50 years [2]–[4]. This rise in pulse pressure with advancing age is consistent with an increase in large artery stiffness [5] leading to a larger forward pressure wave [3]. The pressing question is then why the autonomic nervous system, which controls blood pressure through modulating vascular resistance, blood volume (through renal function) and cardiac output [6], [7], does not compensate for the increase in pulsatile load following stiffening of the arterial wall.

Because the arterial baroreceptors do not respond to pressure, but to strain [6], we hypothesized that the stiffening of the arterial wall [8], [9] may lead to constitutively reduced signaling from the baroreceptors to the barosensitive sympathetic efferents [6] at high pulse pressure. By misinforming the autonomic nervous system about the actual blood pressure and thus preventing it from exerting a proper negative feedback response through regulation of the heart rate, vasculature and renal system, the compromised baroreceptor function then hypothetically leads to an increasing baseline pulse pressure with increasing stiffening of the aortic wall.

**Figure 1:**
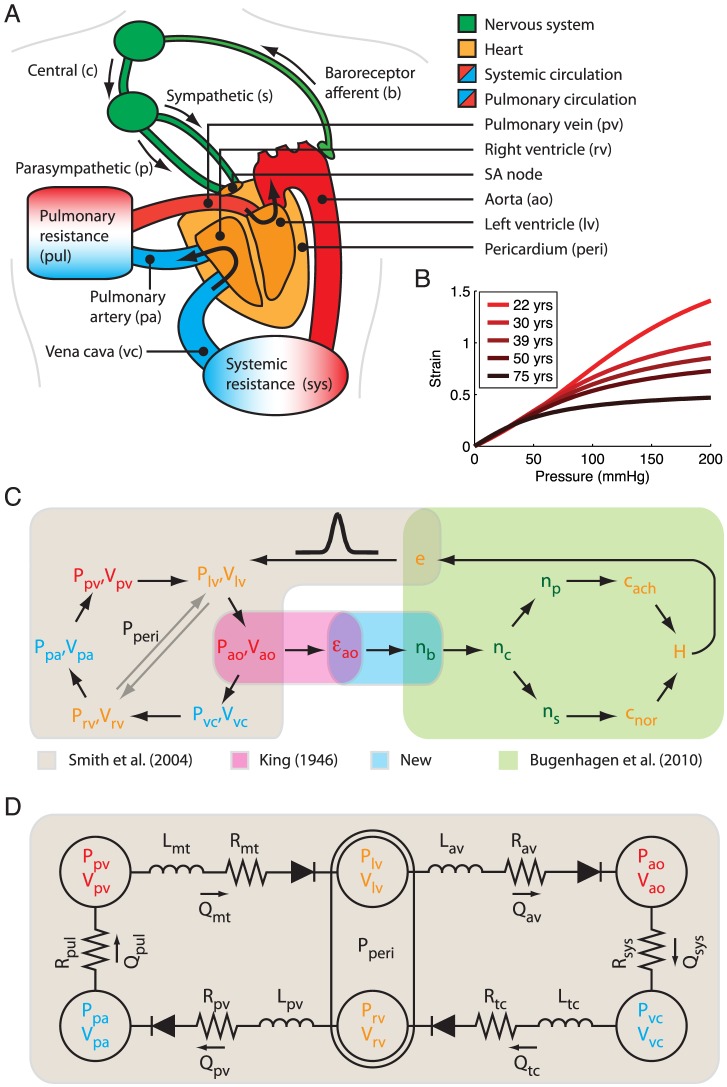
Model overview. (A) Schematic illustration of the anatomical structures contained in the model. (B) Strain-pressure relationships for various age groups [15], [18] used in the integrated model. (C) Model variables and their interconnections: 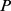 is pressure; 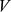 is volume; 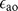 is aortic wall transversal strain; 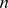 is firing rate; 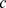 is concentration; 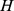 is beat driver; 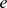 is the beat driver function, which produces heart beats through the dynamic contribution to the pressure-volume relationships of the heart chambers. (D) Circuit diagram of the cardiovascular model: Q is flow rate; R is resistance; L is inductive inertial effects [11].

Our hypothesis does not challenge currently accepted mechanisms for blood pressure regulation by the renal system [10]. However, because information about actual blood pressure to the renal system is conveyed through the sympathetic system based on baroreceptor response to strain, it implies that the increase in sympathetic tone associated with increasingly more dysfunctional baroreceptor signaling with age shifts the renal pressure-diuresis/natriuresis function curve to higher pressures. To demonstrate the viability of our hypothesis in quantitative terms we integrated age-dependent arterial stiffening into a composite circulatory and baroreflex model in which the sympathetic and parasympathetic nervous activity regulate the heart rate in response to changes in blood pressure (Fig. 1).

The lumped parameter model (see Methods for a detailed description) is based on the circulatory model by Smith et al. [11], [12], the baroreflex model of Bugenhagen et al. [13], [14], a model of the age-dependent aortic strain-pressure relationship established by Allen L. King in 1946 [15] (Fig. 1B), and a baroreceptor stimulus-response model following from standard receptive field theory of neurons [16]. Guided by experimental data [17] we included adaptation of the baroreceptors through changes in the baroreceptor thresholds and gains for the various age groups. By assuming a constant blood volume for all age groups, the model explicitly does not account for the regulation of plasma volume and salt through the kidney and the renin-angiotensin system [10] following from the hypothesized shift in the renal pressure-diuresis/natriuresis function curve. Neither did we include any other adaptive change in the heart or the vasculature that could partially ameliorate the effects of arterial stiffening on blood pressure. The exclusion of such partially compensating mechanisms, which most likely exist, was deliberately done to test the explanatory sufficiency of a mechanogenic mechanism with regard to the emergence of primary hypertension with age. The rationale being that by establishing such sufficiency our model framework would provide a sound foundation for systematically incorporating and quantify the effects of these compensatory mechanisms later on.

Our mechanogenic hypothesis explains why the kidneys do not restore blood pressure to normal levels with arterial stiffening. By this it subsumes more of the biology involved in the etiology of hypertension compared to a renogenic or renocentric explanation and provides a new interpretational framework for available experimental and clinical data. This conceptual advance is likely to provide guidance for further experimental and theoretical work as well as drug development. Several authors have pointed to the possible etiological importance of reduced aortic compliance with age in relation to hypertension, see [7] and references therein. However, this is the first clear quantitative demonstration that arterial stiffening is sufficient to explain primary hypertension. And in terms of a high-level phenotype involving the concerted action of several organ systems, this paper demonstrates the need for accounting for the physiology of the ageing phenotype in quantitative terms when we seek to understand the etiology of complex diseases.

## Results

**Figure 2:**
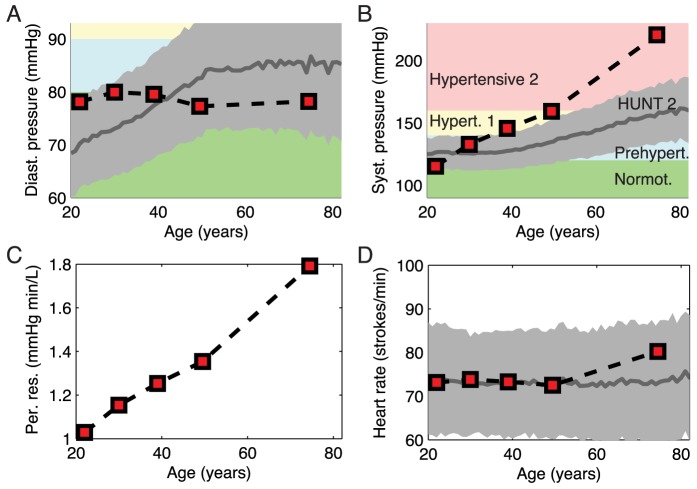
Predicted age-dependent central hemodynamics. Steady state values of (A) diastolic pressure, (B) systolic pressure, (C) peripheral resistance and (D) heart rate, obtained by assuming an age-dependent cardiac output (see main text). The squares refer to the age groups depicted in Fig. 1B. The solid grey lines show recorded mean values (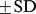 in grey) from 62496 individuals in the age range 20–80 years obtained from the Norwegian HUNT2 Survey [22]. When assessing the fit between predicted and experimental data it should be emphasized that the HUNT2 data also include all individuals (8396) that were under antihypertensive therapy. The blood pressure categorization in panels A and B, from Normotensive (green) to Hypertensive 2 (red), is based on the JNC 7 classification [23].

The consequences of arterial stiffening on cardiovascular function were simulated for various age groups based on the aortic strain-pressure relationship that follows from increased stiffening of the arterial wall [15], [18] (Fig. 1B). Making use of the empirical observation that mean cardiac output falls with about 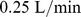 per decade [19], [20], and assuming a baseline value of about 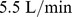 for the youngest age group [21], enabled us to predict an approximately 1.75× linear increase of peripheral resistance across the focal age range (Fig. 2C). This relationship was then used to constrain the baseline peripheral resistance when calculating the central hemodynamic characteristics for the various age groups.

Without changing any heart-specific parameters, the model predicts an almost linear decrease in stroke volume with age. Its predictions concerning temporal development of mean diastolic pressure and systolic pressure are concordant with available empirical data from the Framingham study [5] and the Norwegian HUNT2 Survey counting 62500 individuals [22] (Fig. 2A, B, D). While the diastolic pressure for all individuals is predicted to be categorized as normotensive or slightly prehypertensive according to the JNC-7 classification [23] (Fig. 2A (rectangles)), the systolic pressures of the three oldest age groups are predicted to be in the stage 1 hypertensive and severely stage 2 hypertensive groups. While the trend of increasing systolic pressure with age emerges from the simulations, model predictions overestimate values of systolic pressure as expected. However, the discrepancy is particularly large for the oldest group (71–78 yr) [1]–[4].

**Figure 3:**
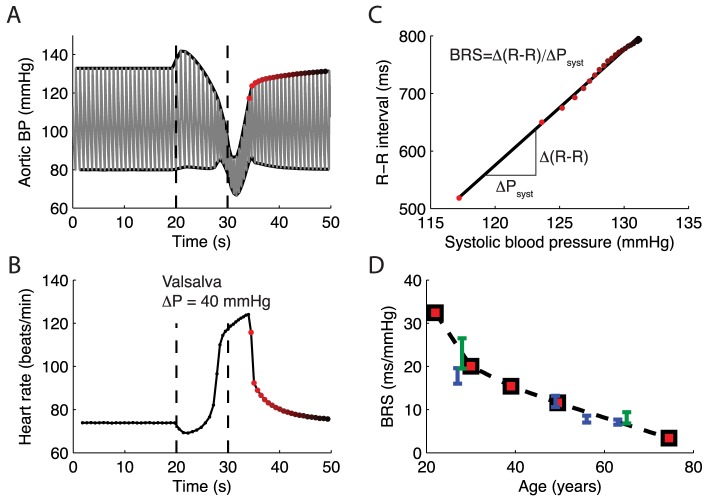
Model response to Valsalva maneuver. (A) Predicted changes in aortic blood pressure and (B) heart rate following from exposing individuals around 30 years to the Valsalva maneuver. The maneuver was mimicked by an increase in thoracic pressure of 40 mmHg, starting at 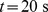 and lasting for 10 seconds. (C) The baroreflex sensitivity (BRS) is computed by finding the slope of 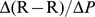 within the time interval just after the heart rate in B has reached its peak, indicated by the red dots in A and B, which are bright just after the Valsalva maneuver and then turn dark. The R—R interval is given from the inverse of the heart rate in B. (D) Comparison between predicted BRS values for all ages and available experimental data, green shows data for sedentary men from [47] and blue shows data for sedentary men from [48].

The model predictions are dependent on the stipulated relationship between arterial distensibility and baroreflex signaling (i.e. baroreflex sensitivity (BRS)), obtained from combining the age-dependent aortic volume-pressure relationship developed by King [15] with our baroreceptor stimulus-response model based on standard receptive field theory of neurons [16]. We tested the predicted relationship between BRS and age by first mimicking a standard Valsalva maneuver (i.e. inducing a brief temporal increase in thoracic pressure) on a young individual. Confirming that the model was indeed capable of predicting major features of the Valsalva maneuver in a young normotensive individual (Fig. 3A, B, C), we then used the in silico Valsalva maneuver to extract the BRS values for all age groups. The model results agree nicely with experimental data showing that cardiovagal baroreflex sensitivity declines progressively with age and is positively related to carotid artery compliance [7], [24] (Fig. 3D). Using these experimental data as test data instead of calibration data enabled us to make an independent assessment of a critical underlying premise of the integrated model.

**Figure 4:**
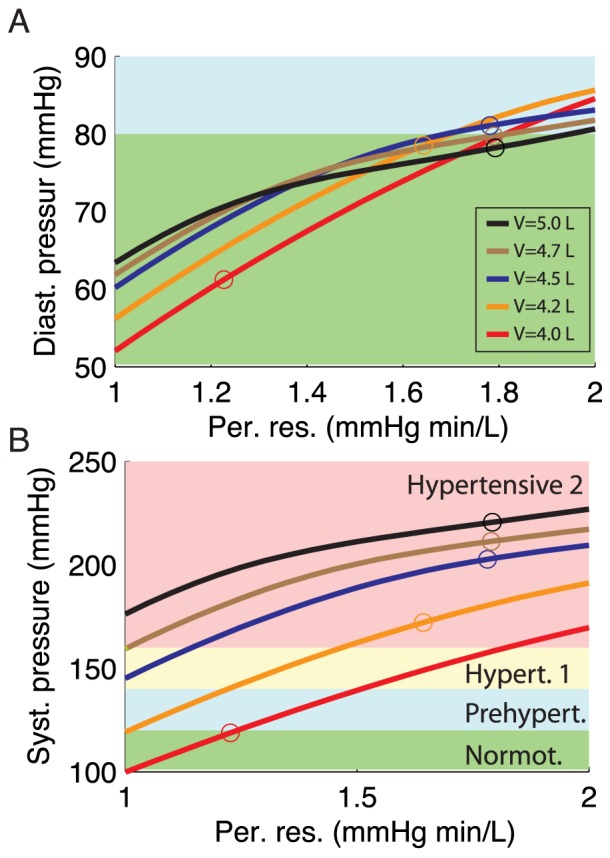
Effect of reducing blood volume and peripheral resistance for the oldest age group (75 years). The five curves show results for different blood volumes, and the background colors indicate blood pressure categories (see Legend to Fig. 2). For both panels the five curves correspond to a blood volume of 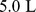, 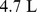, 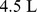, 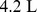 and 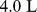, as indicated by the legend in A. The circles show the blood pressures and the corresponding peripheral resistances for the default cardiac output for the oldest age group [19], [20]. (A) Diastolic aortic pressure. (B) Systolic aortic pressure.

The mechanogenic hypothesis is intimately related to the fact that the baroreceptors do not respond to changes in blood pressure, but to changes in strain, and thus are likely to misinform the sympathetic system about the actual state of affairs when located in less compliant vessels. Recent experiments [25], demonstrating that the generally observed drop in blood pressure that follows from chronic stimulation of the carotid baroreflex can partly be attributed to sustained inhibition of renal sympathetic nerve activity, strongly supports this interpretation. It is also supported by data from renal denervation experiments [26], [27], which suggest that the sympathetic regulation of the kidneys, whether it is correctly informed or misinformed about the actual blood pressure by the baroreceptors, prevents activation of alternative regulatory mechanisms that apparently become invoked after denervation. It seems likely that these mechanisms at least in part cause a reduction in peripheral vascular resistance and/or blood volume [6], [28], two of the parameters in our model. Assuming normal renal function, the model predicts that even a moderate reduction in these parameters, due to diminished influence from a misinforming sympathetic control regime, may lead to the experimentally observed drop in pulse pressure of about 20 mmHg six months post treatment [26], [27] (Fig. 4).

## Discussion

Our model-based analysis allows us to probe the potential influence of the arterial stiffening and the baroreflex system in isolation from the influence of other regulatory mechanisms influencing sympathetic activity beyond the baroreflex [10]. Thus this analysis reveals that this mechanism on its own may explain the emergence of hypertension with age. By not including any compensation mechanism through adaptive changes in the heart or the vasculature to increase in blood pressure, we consider the discrepancy between model predictions and empirical data in Fig. 2 to strengthen the case for our mechanogenic hypothesis. If the model had predicted a weaker relation between blood pressure increase and age than empirically observed, this would have made the predominance of a mechanogenic mechanism much less likely. On the other hand, the empirical data on age-related hypertension clearly imply that compensatory mechanisms are able to only partially ameliorate the effects of arterial stiffening on blood pressure. Indeed, the first step towards quantifying the effects of such partial compensation by the kidneys was recently made by Beard et al. [29]. However, their model needs to be further integrated with the one presented here in order to predict the effects of the counteraction by the kidneys to the chronic change in sympathetic tone associated with reduced arterial compliance. If this counteraction as a function of age-related change in sympathetic tone turns out to be highly nonlinear, the observed increased discrepancy for the two oldest age groups in Fig. 2 may be accounted for without introducing any additional explanatory mechanism. However, it should also be noted that despite assuming fully functional baroreceptors, our model predicts that there is still almost no baroreflex signaling with changing blood pressure for this age group. Assuming this stands up to test, an alternative (or additional) explanation of the observed discrepancy is that the effects of this almost total loss of excitatory input from baroreceptor afferents may be similar to those suggested to emerge from sinoaortic denervation (SAD) [30]–[33], i.e. a reorganization of the neural activity within the nucleus of the solitary tract (NTS) [33] and perhaps other nuclei in the baroreflex pathway that lead to a more inhibited sympathetic outflow among elderly with autonomous nervous systems experiencing strong to almost total loss of excitatory input from the baroreceptor afferents.

According to Guytons model of blood pressure regulation [10], any long-lasting alteration in blood pressure requires a shift of the kidneys acute pressure-natriuresis relationship (PNR). While this standard model acknowledges that renal dysfunction need not be the primary event in the cascade of changes leading to hypertension, it is asserted that whatever is the primary cause, it must lead to a change in the kidneys' ability to excrete salt and water at a given level of blood pressure [34]. There is no conflict between this assertion and our results. But our analysis shows that we do not need to invoke any pathophysiological change in kidney function, or to include a specific model of the renal system, to explain the emergence of hypertension with age. On the other hand, it is well documented that chronic elevation of renal perfusion pressure results in renal injury (arteriolar wall thickening, glomerular sclerosis and tubular sclerosis and interstitial sclerosis) [35]. Thus, as renal function is diminished by pressure-induced injury, our model is fully concordant with the possibility that this may over time cause further elevation of blood pressure. Such a possible detrimental positive feedback loop points to the clinical fact that frequently there is a temporality associated with the etiology of a pathological condition, i.e. the underlying causes do not necessarily emerge or operate independently of each other.

A frequently stated argument against baroreceptor participation in determining blood pressure level is that they adapt to the prevailing pressure over time [36] and thus, cannot provide a sustained error signal to reflex mechanisms controlling the sympathetic nervous system. Based on a mechanogenic hypothesis our model is entirely consistent with observations on the resetting of baroreflex sensitivity in primary hypertension [37], and provides at least partial explanation for the phenomenon. The interpretation revealed by our analysis is that resetting, caused at least in part by mechanical remodeling of arteries, represents a primary cause rather than a consequence of hypertension. This is strongly supported by a comprehensive recent study by Kaess et al. [38] concluding that vascular stiffness appears to be a precursor rather than the result of hypertension. Furthermore, it has been shown that renal dysfunction observed in the Dahl SS rat model of hypertension may be explained as resulting primarily from stiffening of renal arterioles [39], consistent with the overall hypothesis explored here. If hypertension causes arterial stiffening, the resulting positive feedback loop between arterial stiffening and hypertension does not challenge our mechanogenic hypothesis. Moreover, as the underlying causes of arterial stiffening is not explicitly modelled, the current model captures such a mechanism very well.

One may argue that our results cannot be reconciled with the longstanding observation that sinoaortic denervation (SAD) does not necessarily produce constant fixed hypertension. However, the interpretation of those observations is complex and controversial for many reasons. First, while numerous studies show that SAD does not lead to fixed hypertension, there certainly exist studies showing that it does (e.g. Machado and Brady [40], Rodrigues et al. [41]). There is, however, consensus that SAD tends to cause a marked increase in pressure within the first hours/days. Thus it is clear that ablation of the baroreflex has an effect on pressure over the time scale of hours/days, but that the effect in several cases seems to go away over the timescale of weeks. Thus, our predictions are in agreement with the increase in pressure observed in the timescale of hours/days following SAD, but do not capture what happens at the time scale of weeks after SAD. Regardless, we do not necessarily expect our model to capture either phase of this effect as it was not designed to account for an experiment in which the afferent nerves are severed. Under our mechanical hypothesis, a misrepresentation of the pressure is delivered by the baroreceptor afferents, which leads to a chronic increase in pressure. When the afferent nerve is severed, the signal is ablated. Thus, we do not necessarily expect the central nervous system to respond to the removal of an input signal in the same manner as if the signal misrepresents the state of the system.

Another possible concern is that baroreceptor dysfunction is not required to produce hypertension in the model given that aortic stiffness for example increases 3–4× more than stroke volume falls. Note, however, that our mechanogenic hypothesis assumes fully functional baroreceptors as such. As the baroreceptors respond to strain and not pressure, the dysfunction appears when aortic strain, due to stiffening, no longer is a good proxy for aortic blood pressure. In the model the aortic stiffness causes hypertension because the blood volume and peripheral resistance are not regulated optimally, as the nervous system (and therefore the kidneys) is misinformed about the blood pressure by the baroreceptors. The regulatory system would probably have been able to handle rather high arterial stiffness if the nervous system was given correct information about blood pressure from the baroreceptors (Fig. 4). Thus baroreceptor dysfunction in our paper is per definition connected to increase in aortic stiffness.

In agreement with experiments the model results are based on an age-dependent decay in cardiac output from 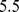 to 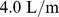 across 6 decades [19], [20] and that peripheral resistance accordingly increases linearly by 75% across the same age range. A possible concern is that this effect alone will guarantee the observed development of hypertension. However, in our model the peripheral resistance is, for each age group, optimized to give a cardiac output in agreement with experimental results on the decline in cardiac output with age [19], [20]. Thus the linear increase in peripheral resistance with age is not an assumption, but a prediction.

One may also argue that aortic stiffening outside the baroreceptor region may very well increase central blood pressure simply because of increased pulse wave velocity. However, if this were the case, our model predicts that with intact baroreceptor function, the regulatory machinery under control of the sympathetic system will to a considerable degree compensate for this aortic stiffening. Fig. 4 shows how moderate changes in blood volume and peripheral resistance may compensate for an increase in systolic pressure due to aortic stiffening.

A lumped parameter model is per definition a model that simplifies the description of the behavior of a spatially distributed physical system into a topology consisting of discrete entities that approximates the behavior of the distributed system under certain assumptions. Considering the concordance between model predictions and empirical data, the resolution level of our model appears appropriate for what we set out to test in this paper. However, the predictions in our study might benefit from utilizing a mathematical approach which accounts for wave propagation effects (e.g. 1-D network models for compliant vessels). In such a case one could investigate the effect of the regional changes in compliance on the timing of the reflected waves, as reflections which reach the aortic valve and the left ventricle before closure, will pose a greater load to the heart, whereas reflected waves which arrive at the aortic valve after the valve closure will act as a positive agent for cardiac perfusion. The construction of 3-D fluid-solid interaction models to describe both the local changes in hemodynamic loads and wall strain is an ultimate goal though. However, before one can make reliable models one would have to harvest vast amounts of material data (e.g. pulse wave velocities) for all the vessels incorporated in the model. Such data appear to be quite daunting to collect with current technology, but as our model suggests that the aortic wall strain and baroreceptor output are key factors in blood pressure regulation, it strongly motivates the generation of appropriate technology. This would also most likely lead to a better understanding of the etiology of hypertension at the individual level.

Our analysis illustrates the clear need for accounting for the ageing phenotype in efforts to understand the etiology of complex diseases. As the baroreceptors respond to strain and not pressure, the blood pressure regulatory system becomes dysfunctional when aortic strain, due to age-related stiffening, is no longer a good proxy for aortic blood pressure. The lack of mechanisms that fully compensate for the increasing aortic stiffness with age can easily be explained by standard evolutionary theory of aging [42].

Finally, our results suggest that arterial stiffness represents a therapeutic target by which we may be able to exploit an otherwise intact machinery for integrated blood pressure regulation.

## Materials and Methods

### Model overview

Our model is a composite of the circulatory model of Smith et al. [11] and the baroreflex model of Bugenhagen et al. [13], with modified heart dynamics, a new receptive field model for the baroreceptor stimulus-response relationship [16] and the King model of the aorta dynamics based on the age-dependent and nonlinear volume-pressure relationship derived from basic physical principles of elastomers [15]. The full model can be downloaded from http://virtualrat.org/computational-models/vpr1003/.

Parameter values in the baroreflex model were set to original values reported in Bugenhagen et al. [13] for all components representing processes of the central nervous system activity, the dynamics of norepinephrine and acetylcholine at the sinoatrial (SA) node of the heart, and the effects of these concentrations on heart rate. For the unmodified parts of the cardiovascular system the original parameter values for the Smith et al. model were used. Below we focus on the novel model elements. One should consult original references [11], [13], [15] for the parts of the model that were not modified.

### Heart and circulatory system

The original Smith et al. heart and circulatory model [11], which simulates cardiac pumping at a constant heart rate 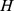, was modified to use a variable input heart rate driving function 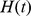, which is determined by the baroreflex model. The function 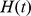, which depends on model-simulated acetylcholine and norepinephrine concentrations [13], is a continuous function of time 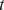 and thus in general varies within one heart cycle. The complexity of the heart model was reduced by removal of the septum compartment, as the results with and without septum were indistinguishable by eye (see supplementary Fig. S1 for quantitative differences for the default parameters used in Smith et al. [11]), and removal of the septum caused the Matlab programme to run much more efficiently. A mechanical interaction between the heart ventricles remained trough the shared pericardium volume.

The cardiac domain contractilities/elastances are assumed to vary in proportion to the heart rate,
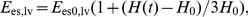
(1)

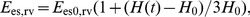
(2)where a 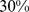 decrease in heart rate gives a 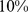 decrease in elastance [43]. The subscripts ‘lv’ and ‘rv’ denote the left ventricle and right ventricle, respectively. A constant value of 
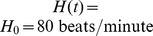
 gives the default elastance valus 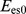 [11].

The linear pressure-volume relationships used in Smith et al. [11] are independent of the total blood volume and the model thus considers only the *stressed blood volumes* (with a total stressed blood volume of 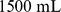). Here, we used the non-linear pressure-volume relationship from King [15], and we assumed a total blood volume of 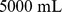. In the King model the pressure is given by a non-linear function of the relative volume 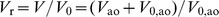,
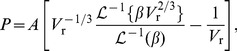
(3)where 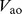 is the stressed aortic volume, 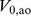 is the age-dependent unstressed aortic volume, 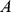 and 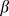 are age-dependent parameters from King [15], 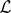 is the Langevin function,
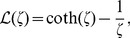
(4)and 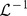 is the inverse Langevin function. The inverse Langevin function poses analytical challenges. However, within the domain of validity, 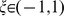, the inverse Langevin function is well approximated with less than 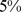 error at any point [44] by
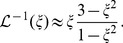
(5)We therefore made consequent use of this approximation in our model.

In the King model the aorta is approximated as a cylinder: the aortic resting volume is given by 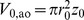 and the stressed volume is given by 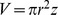, with 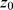 and 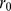 as the non-stressed and 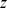 and 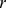 as the stressed lengths and radiuses of the aortic cylinder, respectively. Further, the aortic wall is assumed to be perfectly elastomeric with the relationship between the length 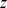 and radius 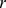 given by 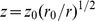. It then follows that the pressure can be expressed equivalently by the relative quantities 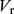, 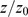 or 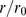. In the King model the zero-pressure reference volume corresponding to 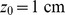 is used, but in the present formalism the reference volume was chosen to give stressed volumes roughly in agreement with the original stressed volumes of the Smith model, which is about 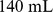 at a pressure of 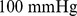. This was achieved by setting 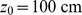 for all ages. For an aortic pressure of 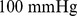 this gives total aortic volumes in the range from 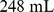 (youngest) to 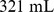 (oldest) and stressed aortic volumes in the range from 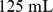 (oldest) to 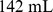 (youngest) for the different ages.

### Baroreceptor afferent

The relative volume 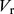 is related to the aortic radius [15], 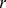, through
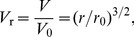
(6)where 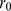 is the non-stressed aortic radius. By using the definition of the strain,
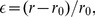
(7)Eq. 3 gives the pressure-strain relationship
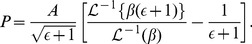
(8)


In our model the strain is the input stimulus, to which the baroreceptor responds with a given firing rate. A linear stimulus-response model was constructed by expressing the linear firing rate 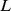 as a convolution of the stimulus,
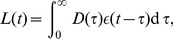
(9)where 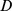 is the temporal kernel relating the stimulus to the response. A static nonlinearity function 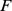 was introduced to model the firing-rate threshold. The non-linear firing rate, denoted 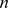, can then be expressed as
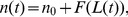
(10)where 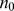 is the background firing rate and 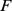 is the linear threshold function [16],
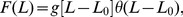
(11)

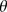
 is the Heaviside step function, 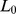 is the threshold value that 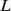 must overcome to start firing, and 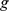 is a proportionality constant. Fig. 4A in Bugenhagen et al. [13], which is a reproduction of experimental results from Brown et al. [45], shows that experimentally induced steps in blood pressure give sharp overshoots of firing rate, followed by much slower saturations. Such an overshoot followed by a saturation can be modeled with a linear kernel 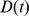 consisting of the two-exponential function,
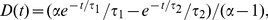
(12)with time constants 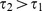. The kernel 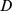 is normalized so that the convolution integral, Eq. 9, gives 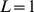 for stimulus 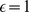. The parameters 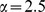, 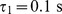 and 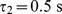 of Eq. 12 were found to give temporal responses to pressure step functions similar to the experiments.

In Andresen et al. [17] the gain 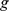 and threshold 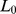 are shown to express adaptation to increased stiffness of the aortic wall. In their Fig. 5B two rats with different aortic stiffnesses are shown to express very different pressure-strain relationships, and their Fig. 7C shows adaptation in the corresponding firing rate responses. The pressure-strain curves in their Fig. 5B resemble the pressure-strain curves for ages 39 years and 75 years reported by King [15], and the corresponding firing rate thresholds and gains, 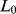 and 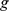, were therefore used as thresholds and gains for the corresponding ages: 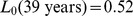, 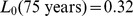, 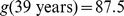 and 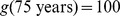. For the other ages the parameters for threshold and gain were intra- and extrapolated from these values.

The convolution formalism is tractable if the baroreflex is modeled as an open-loop process, which is not possible here as the baroreflex is part of a closed-loop system in which pressure influences the baroreflex afferent tone and the baroreflex efferent tone influences the pressure. Since the convolution kernel is expressed by decaying exponential functions, the convolution can, however, be transferred to equivalent differential equations [46]. It can be shown [46] that the convolution integral given in Eq. 9 with an exponential kernel 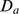,
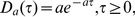
(13)can be equivalently expressed by
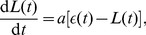
(14)with the initial conditions,
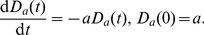
(15)In our model the convolution integral can be split into two terms,
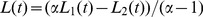
(16)with
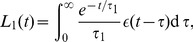
(17)and
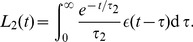
(18)The corresponding differential equations will then be
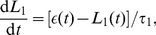
(19)and
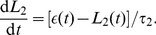
(20)Thus, given the stimulus 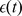, 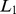 and 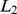 are determined from Eqs. 19 and 20. Overall response 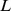 is computed from Eq. 16 and the baroreceptor firing rate 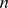 is given by Eq. 10.

## Supporting Information

Figure S1 **Comparison of the Smith model and a similar model without septum.** The Smith model [11] (thin blue line) is identical to the model without septum (thick red line) except for the septum volume dynamics: for the model without septum the septum volume is set to zero. (A) Right ventricular pressure, (B) left ventricular pressure, (C) aortic pressure. The respective pressure differences between the two models are shown in panels D–F. All pressures and pressure differences are expressed in units of mmHg.
(EPS)
